# Diaqua­bis(4-methyl­benzoato-κ*O*)bis­(nicotinamide-κ*N* ^1^)nickel(II)

**DOI:** 10.1107/S1600536810007385

**Published:** 2010-03-03

**Authors:** Hacali Necefoğlu, Efdal Çimen, Barış Tercan, Emel Ermiş, Tuncer Hökelek

**Affiliations:** a Department of Chemistry, Kafkas University, 36100 Kars, Turkey; b Department of Physics, Karabük University, 78050 Karabük, Turkey; c Department of Chemistry, Faculty of Science, Anadolu University, 26470 Yenibağlar, Eskişehir, Turkey; d Department of Physics, Hacettepe University, 06800 Beytepe, Ankara, Turkey

## Abstract

The title Ni^II^ complex, [Ni(C_8_H_7_O_2_)_2_(C_6_H_6_N_2_O)_2_(H_2_O)_2_], is centrosymmetric with the Ni atom located on an inversion center. The mol­ecule contains two 4-methyl­benzoate (PMB) and two nicotinamide (NA) ligands and two coordinated water mol­ecules, all ligands being monodentate. The four O atoms in the equatorial plane around the Ni atom form a slightly distorted square-planar arrangement, while the slightly distorted octa­hedral coordination is completed by the two N atoms of the NA ligands in the axial positions. The dihedral angle between the carboxyl­ate group and the adjacent benzene ring is 26.15 (10)°, while the pyridine and benzene rings are oriented at a dihedral angle of 87.81 (4)°. In the crystal structure, inter­molecular O—H⋯O and N—H⋯O hydrogen bonds link the mol­ecules into a three-dimensional network. The π–π contact between the benzene rings [centroid–centroid distance = 3.896 (1) Å] may further stabilize the crystal structure. A weak C—H⋯π inter­action involving the pyridine ring also occurs.

## Related literature

For niacin, see: Krishnamachari (1974 ▶) and for the nicotinic acid derivative *N*,*N*-diethyl­nicotinamide, see: Bigoli *et al.* (1972 ▶). For related structures, see: Hökelek *et al.* (1996 ▶, 2009*a* ▶,*b* ▶,*c* ▶); Hökelek & Necefoğlu (1998 ▶).
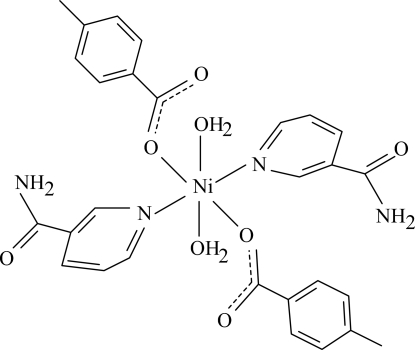


## Experimental

### 

#### Crystal data

[Ni(C_8_H_7_O_2_)_2_(C_6_H_6_N_2_O)_2_(H_2_O)_2_]
*M* *_r_* = 609.26
Triclinic, 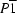

*a* = 7.7324 (2) Å
*b* = 9.7335 (3) Å
*c* = 9.8198 (3) Å
α = 78.440 (2)°
β = 86.475 (3)°
γ = 71.662 (2)°
*V* = 687.31 (4) Å^3^
*Z* = 1
Mo *K*α radiation
μ = 0.76 mm^−1^
*T* = 99 K
0.33 × 0.28 × 0.25 mm

#### Data collection

Bruker Kappa APEXII CCD area-detector diffractometer
Absorption correction: multi-scan (*SADABS*; Bruker, 2005 ▶) *T*_min_ = 0.889, *T*_max_ = 0.934
12002 measured reflections
3390 independent reflections
3034 reflections with *I* > 2σ(*I*)
*R*_int_ = 0.024

#### Refinement

*R*[*F* ^2^ > 2σ(*F* ^2^)] = 0.030
*wR*(*F* ^2^) = 0.068
*S* = 1.05
3390 reflections
204 parameters
H atoms treated by a mixture of independent and constrained refinement
Δρ_max_ = 0.48 e Å^−3^
Δρ_min_ = −0.50 e Å^−3^

### 

Data collection: *APEX2* (Bruker, 2007 ▶); cell refinement: *SAINT* (Bruker, 2007 ▶); data reduction: *SAINT*; program(s) used to solve structure: *SHELXS97* (Sheldrick, 2008 ▶); program(s) used to refine structure: *SHELXL97* (Sheldrick, 2008 ▶); molecular graphics: *ORTEP-3 for Windows* (Farrugia, 1997 ▶); software used to prepare material for publication: *WinGX* (Farrugia, 1999 ▶) and *PLATON* (Spek, 2009 ▶).

## Supplementary Material

Crystal structure: contains datablocks I, global. DOI: 10.1107/S1600536810007385/xu2730sup1.cif

Structure factors: contains datablocks I. DOI: 10.1107/S1600536810007385/xu2730Isup2.hkl

Additional supplementary materials:  crystallographic information; 3D view; checkCIF report

## Figures and Tables

**Table 1:** Selected bond lengths (Å)

Ni1—O1	2.0621 (10)
Ni1—O4	2.0870 (10)
Ni1—N1	2.0859 (12)

**Table 2:** Hydrogen-bond geometry (Å, °) *Cg*2 is the centroid of the N1/C9–C13 ring.

*D*—H⋯*A*	*D*—H	H⋯*A*	*D*⋯*A*	*D*—H⋯*A*
N2—H21⋯O2^i^	0.86 (2)	2.037 (19)	2.8333 (18)	153.4 (19)
N2—H22⋯O3^ii^	0.90 (2)	2.05 (2)	2.9192 (19)	161.5 (18)
O4—H41⋯O3^iii^	0.81 (2)	2.10 (2)	2.8864 (16)	162.9 (19)
O4—H42⋯O2^iv^	0.89 (2)	1.75 (2)	2.6240 (16)	165 (2)
C6—H6⋯*Cg*2^v^	0.93	2.65	3.5737 (18)	171

Symmetry codes: (i) 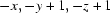; (ii) 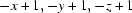; (iii) 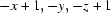; (iv) 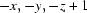; (v) 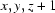.

**Fig. 1.**
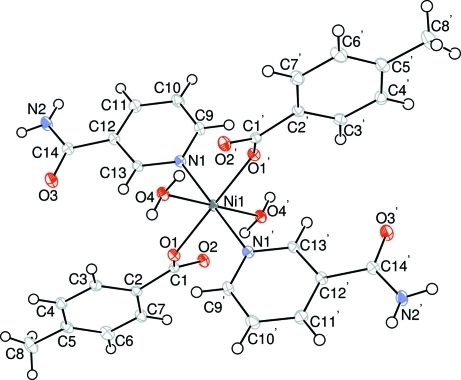
The molecular structure of the title molecule with the atom-numbering scheme. Displacement ellipsoids are drawn at the 20% probability level. Primed atoms are generated by the symmetry operator:(') -x, -y, 1 - z.

## supplementary crystallographic information

### Comment

As a part of our ongoing investigation on transition metal complexes of nicotinamide (NA), one form of niacin (Krishnamachari, 1974), and/or the nicotinic acid derivative *N*,*N*-diethylnicotinamide (DENA), an important respiratory stimulant (Bigoli *et al.*, 1972), the title compound was synthesized and its crystal structure is reported herein.

The title complex, (I), is a crystallographically centrosymmetric mononuclear complex, consisting of two nicotinamide (NA) and two 4-methylbenzoate (PMB) ligands and two coordinated water molecules. The crystal structures of similar complexes of Cu^II^, Co^II^, Ni^II^, Mn^II^ and Zn^II^ ions, [Cu(C_7_H_5_O_2_)_2_(C_10_H_14_N_2_O)_2_], (II) (Hökelek *et al.*, 1996), [Co(C_6_H_6_N_2_O)_2_(C_7_H_4_NO_4_)_2_(H_2_O)_2_], (III) (Hökelek & Necefoğlu, 1998), [Ni(C_7_H_4_ClO_2_)_2_(C_6_H_6_N_2_O)_2_(H_2_O)_2_], (IV) (Hökelek *et al.*, 2009a), [Mn(C_7_H_4_ClO_2_)_2_(C_10_H_14_N_2_O)_2_(H_2_O)_2_], (V) (Hökelek *et al.*, 2009b) and [Zn(C_7_H_4_BrO_2_)_2_(C_6_H_6_N_2_O)_2_(H_2_O)_2_], (VI) (Hökelek *et al.*, 2009c) have also been reported. In (II), the two benzoate ions are coordinated to the Cu atom as bidentate ligands, while in the other structures all ligands being monodentate.

The title complex, [Ni(PMB)_2_(NA)_2_(H_2_O)_2_], has a centre of symmetry and Ni^II^ ion is surrounded by two PMB and two NA ligands and two water molecules (Fig. 1). All ligands are monodentate. The four O atoms (O1, O4, and the symmetry-related atoms, O1', O4') in the equatorial plane around the Ni atom form a slightly distorted square-planar arrangement, while the slightly distorted octahedral coordination is completed by the two N atoms of the NA ligands (N1, N1') in the axial positions (Fig. 1).

The near equality of the C1—O1 [1.2678 (17) Å] and C1—O2 [1.2654 (17) Å] bonds in the carboxylate group indicates a delocalized bonding arrangement, rather than localized single and double bonds. The average Ni—O bond length is 2.0746 (10) Å (Table 1) and the Ni atom is displaced out of the least-squares plane of the carboxylate group (O1/C1/O2) by 0.5087 (1) Å. The dihedral angle between the planar carboxylate group and the benzene ring A (C2—C7) is 26.15 (10)°, while that between rings A and B (N1/C8—C12) is 87.81 (4)°.

In the crystal structure, intermolecular O—H···O and N—H···O hydrogen bonds (Table 2) link the molecules into a three-dimensional network, in which they may be effective in the stabilization of the structure. The π–π contact between the benzene rings, Cg1—Cg1^i^, [symmetry code (i): 1 - x, -y, 2 - z, where Cg1 is the centroid of ring A (C2—C7)] may further stabilize the structure, with centroid-centroid distance of 3.896 (1) Å. There also exists a weak C—H···π interaction involving the pyridine ring (Table 2).

### Experimental

The title compound was prepared by the reaction of NiSO_4_.6(H_2_O) (1.32 g, 5 mmol) in H_2_O (30 ml) and NA (1.22 g, 10 mmol) in H_2_O (15 ml) with sodium 4-methylbenzoate (1.36 g, 10 mmol) in H_2_O (300 ml). The mixture was filtered and set aside to crystallize at ambient temperature for one week, giving blue single crystals.

### Refinement

Atoms H21, H22 (for NH_2_) and H41, H42 (for H_2_O) were located in a difference Fourier map and refined isotropically. The remaining H atoms were positioned geometrically with C—H = 0.93 and 0.96 Å, for aromatic and methyl H atoms, respectively, and constrained to ride on their parent atoms, with U_iso_(H) = xU_eq_(C), where x = 1.5 for methyl H and x = 1.2 for aromatic H atoms.

### Crystal data

**Table:**

[Ni(C_8_H_7_O_2_)_2_(C_6_H_6_N_2_O)_2_(H_2_O)_2_]	*Z* = 1
*M**_r_* = 609.26	*F*(000) = 318
Triclinic, *P*1	*D*_x_ = 1.472 Mg m^−^^3^
Hall symbol: -P 1	Mo *K*α radiation, λ = 0.71073 Å
*a* = 7.7324 (2) Å	Cell parameters from 6458 reflections
*b* = 9.7335 (3) Å	θ = 2.8–28.3°
*c* = 9.8198 (3) Å	µ = 0.76 mm^−^^1^
α = 78.440 (2)°	*T* = 99 K
β = 86.475 (3)°	Prism, blue
γ = 71.662 (2)°	0.33 × 0.28 × 0.25 mm
*V* = 687.31 (4) Å^3^	

### Data collection

**Table:**

Bruker Kappa APEXII CCD area-detector diffractometer	3390 independent reflections
Radiation source: fine-focus sealed tube	3034 reflections with *I* > 2σ(*I*)
graphite	*R*_int_ = 0.024
φ and ω scans	θ_max_ = 28.4°, θ_min_ = 2.1°
Absorption correction: multi-scan (*SADABS*; Bruker, 2005)	*h* = −10→10
*T*_min_ = 0.889, *T*_max_ = 0.934	*k* = −12→12
12002 measured reflections	*l* = −12→13

### Refinement

**Table:**

Refinement on *F*^2^	Primary atom site location: structure-invariant direct methods
Least-squares matrix: full	Secondary atom site location: difference Fourier map
*R*[*F*^2^ > 2σ(*F*^2^)] = 0.030	Hydrogen site location: inferred from neighbouring sites
*wR*(*F*^2^) = 0.068	H atoms treated by a mixture of independent and constrained refinement
*S* = 1.05	*w* = 1/[σ^2^(*F*_o_^2^) + (0.0238*P*)^2^ + 0.4565*P*] where *P* = (*F*_o_^2^ + 2*F*_c_^2^)/3
3390 reflections	(Δ/σ)_max_ < 0.001
204 parameters	Δρ_max_ = 0.48 e Å^−^^3^
0 restraints	Δρ_min_ = −0.50 e Å^−^^3^

### Special details

**Table:**

Geometry. All esds (except the esd in the dihedral angle between two l.s. planes) are estimated using the full covariance matrix. The cell esds are taken into account individually in the estimation of esds in distances, angles and torsion angles; correlations between esds in cell parameters are only used when they are defined by crystal symmetry. An approximate (isotropic) treatment of cell esds is used for estimating esds involving l.s. planes.
Refinement. Refinement of *F*^2^ against ALL reflections. The weighted *R*-factor wR and goodness of fit *S* are based on *F*^2^, conventional *R*-factors *R* are based on *F*, with *F* set to zero for negative *F*^2^. The threshold expression of *F*^2^ > σ(*F*^2^) is used only for calculating *R*-factors(gt) etc. and is not relevant to the choice of reflections for refinement. *R*-factors based on *F*^2^ are statistically about twice as large as those based on *F*, and *R*- factors based on ALL data will be even larger.

### Fractional atomic coordinates and isotropic or equivalent isotropic displacement parameters (Å^2^)

**Table:**

	*x*	*y*	*z*	*U*_iso_*/*U*_eq_	
Ni1	0.0000	0.0000	0.5000	0.00996 (8)	
O1	0.12751 (13)	0.01072 (11)	0.67419 (11)	0.0129 (2)	
O2	−0.11125 (14)	0.15789 (11)	0.77116 (11)	0.0149 (2)	
O3	0.44171 (14)	0.33247 (11)	0.48634 (12)	0.0177 (2)	
O4	0.25984 (15)	−0.07089 (12)	0.41538 (12)	0.0136 (2)	
H41	0.342 (3)	−0.137 (2)	0.458 (2)	0.034 (6)*	
H42	0.228 (3)	−0.103 (2)	0.345 (2)	0.036 (6)*	
N1	−0.00159 (16)	0.21391 (13)	0.41144 (13)	0.0116 (2)	
N2	0.33786 (19)	0.55995 (15)	0.35530 (15)	0.0176 (3)	
H21	0.252 (3)	0.628 (2)	0.307 (2)	0.022 (5)*	
H22	0.425 (3)	0.586 (2)	0.390 (2)	0.024 (5)*	
C1	0.05766 (19)	0.09453 (16)	0.75988 (15)	0.0120 (3)	
C2	0.18258 (19)	0.12691 (15)	0.85126 (15)	0.0121 (3)	
C3	0.3601 (2)	0.12032 (16)	0.81040 (16)	0.0139 (3)	
H3	0.4070	0.0851	0.7302	0.017*	
C4	0.4678 (2)	0.16598 (16)	0.88863 (16)	0.0153 (3)	
H4	0.5855	0.1624	0.8593	0.018*	
C5	0.4019 (2)	0.21690 (17)	1.00998 (17)	0.0162 (3)	
C6	0.2262 (2)	0.21841 (18)	1.05279 (17)	0.0192 (3)	
H6	0.1816	0.2488	1.1356	0.023*	
C7	0.1171 (2)	0.17547 (17)	0.97424 (16)	0.0164 (3)	
H7	−0.0006	0.1790	1.0037	0.020*	
C8	0.5187 (2)	0.26752 (19)	1.09414 (19)	0.0239 (4)	
H8A	0.4556	0.3664	1.1063	0.036*	
H8B	0.6314	0.2651	1.0463	0.036*	
H8C	0.5435	0.2033	1.1834	0.036*	
C9	−0.13817 (19)	0.31071 (16)	0.33131 (16)	0.0140 (3)	
H9	−0.2385	0.2823	0.3163	0.017*	
C10	−0.1356 (2)	0.45107 (17)	0.27015 (17)	0.0169 (3)	
H10	−0.2326	0.5157	0.2153	0.020*	
C11	0.0139 (2)	0.49377 (16)	0.29208 (16)	0.0156 (3)	
H11	0.0188	0.5875	0.2521	0.019*	
C12	0.15612 (19)	0.39452 (16)	0.37462 (15)	0.0121 (3)	
C13	0.14248 (19)	0.25568 (16)	0.43189 (15)	0.0115 (3)	
H13	0.2378	0.1888	0.4869	0.014*	
C14	0.32352 (19)	0.42736 (16)	0.40920 (16)	0.0131 (3)	

### Atomic displacement parameters (Å^2^)

**Table:**

	*U*^11^	*U*^22^	*U*^33^	*U*^12^	*U*^13^	*U*^23^
Ni1	0.00972 (13)	0.00810 (13)	0.01255 (14)	−0.00273 (10)	−0.00152 (9)	−0.00267 (10)
O1	0.0132 (5)	0.0108 (5)	0.0144 (5)	−0.0023 (4)	−0.0020 (4)	−0.0036 (4)
O2	0.0120 (5)	0.0152 (5)	0.0175 (6)	−0.0027 (4)	−0.0015 (4)	−0.0050 (4)
O3	0.0162 (5)	0.0107 (5)	0.0257 (6)	−0.0035 (4)	−0.0068 (4)	−0.0016 (5)
O4	0.0114 (5)	0.0124 (5)	0.0163 (6)	−0.0018 (4)	−0.0020 (4)	−0.0038 (5)
N1	0.0122 (6)	0.0100 (6)	0.0128 (6)	−0.0029 (5)	−0.0008 (5)	−0.0035 (5)
N2	0.0172 (7)	0.0113 (6)	0.0254 (8)	−0.0066 (5)	−0.0074 (6)	−0.0003 (6)
C1	0.0134 (7)	0.0098 (7)	0.0124 (7)	−0.0044 (5)	−0.0023 (5)	0.0010 (5)
C2	0.0132 (7)	0.0087 (6)	0.0133 (7)	−0.0021 (5)	−0.0032 (5)	−0.0007 (5)
C3	0.0157 (7)	0.0130 (7)	0.0124 (7)	−0.0033 (6)	−0.0010 (5)	−0.0028 (6)
C4	0.0135 (7)	0.0139 (7)	0.0179 (8)	−0.0040 (6)	−0.0021 (6)	−0.0014 (6)
C5	0.0180 (7)	0.0116 (7)	0.0182 (8)	−0.0024 (6)	−0.0062 (6)	−0.0027 (6)
C6	0.0193 (8)	0.0224 (8)	0.0162 (8)	−0.0028 (6)	−0.0002 (6)	−0.0101 (7)
C7	0.0132 (7)	0.0185 (8)	0.0167 (8)	−0.0030 (6)	−0.0003 (6)	−0.0046 (6)
C8	0.0219 (8)	0.0243 (9)	0.0285 (10)	−0.0062 (7)	−0.0070 (7)	−0.0111 (7)
C9	0.0116 (7)	0.0140 (7)	0.0173 (8)	−0.0039 (6)	−0.0022 (6)	−0.0045 (6)
C10	0.0146 (7)	0.0126 (7)	0.0207 (8)	−0.0008 (6)	−0.0058 (6)	−0.0003 (6)
C11	0.0173 (7)	0.0092 (7)	0.0199 (8)	−0.0040 (6)	−0.0024 (6)	−0.0013 (6)
C12	0.0129 (7)	0.0102 (7)	0.0138 (7)	−0.0028 (5)	−0.0005 (5)	−0.0050 (6)
C13	0.0122 (6)	0.0103 (7)	0.0120 (7)	−0.0025 (5)	−0.0019 (5)	−0.0030 (5)
C14	0.0141 (7)	0.0118 (7)	0.0142 (7)	−0.0032 (6)	0.0000 (5)	−0.0053 (6)

### Geometric parameters (Å, °)

**Table:**

Ni1—O1	2.0621 (10)	C4—C5	1.389 (2)
Ni1—O1^i^	2.0621 (10)	C4—H4	0.9300
Ni1—O4^i^	2.0870 (10)	C5—C6	1.394 (2)
Ni1—O4	2.0870 (10)	C5—C8	1.509 (2)
Ni1—N1	2.0859 (12)	C6—H6	0.9300
Ni1—N1^i^	2.0859 (12)	C7—C2	1.393 (2)
O1—C1	1.2678 (17)	C7—C6	1.383 (2)
O2—C1	1.2654 (17)	C7—H7	0.9300
O3—C14	1.2392 (18)	C8—H8A	0.9600
O4—H41	0.81 (2)	C8—H8B	0.9600
O4—H42	0.88 (2)	C8—H8C	0.9600
N1—C9	1.3421 (19)	C9—C10	1.383 (2)
N1—C13	1.3386 (18)	C9—H9	0.9300
N2—C14	1.3294 (19)	C10—H10	0.9300
N2—H21	0.86 (2)	C11—C10	1.388 (2)
N2—H22	0.89 (2)	C11—H11	0.9300
C1—C2	1.500 (2)	C12—C11	1.388 (2)
C2—C3	1.392 (2)	C12—C13	1.390 (2)
C3—H3	0.9300	C13—H13	0.9300
C4—C3	1.389 (2)	C14—C12	1.500 (2)
			
O1^i^—Ni1—O1	180.0	C5—C4—C3	120.87 (14)
O1—Ni1—O4	86.71 (4)	C5—C4—H4	119.6
O1^i^—Ni1—O4	93.29 (4)	C4—C5—C6	118.33 (14)
O1—Ni1—O4^i^	93.29 (4)	C4—C5—C8	120.73 (14)
O1^i^—Ni1—O4^i^	86.71 (4)	C6—C5—C8	120.93 (14)
O1—Ni1—N1	89.94 (4)	C5—C6—H6	119.4
O1^i^—Ni1—N1	90.06 (4)	C7—C6—C5	121.11 (14)
O1—Ni1—N1^i^	90.06 (4)	C7—C6—H6	119.4
O1^i^—Ni1—N1^i^	89.94 (4)	C2—C7—H7	119.8
O4^i^—Ni1—O4	180.0	C6—C7—C2	120.34 (14)
N1—Ni1—O4	86.75 (4)	C6—C7—H7	119.8
N1^i^—Ni1—O4	93.25 (4)	C5—C8—H8A	109.5
N1—Ni1—O4^i^	93.25 (4)	C5—C8—H8B	109.5
N1^i^—Ni1—O4^i^	86.75 (4)	C5—C8—H8C	109.5
N1^i^—Ni1—N1	180.0	H8A—C8—H8B	109.5
C1—O1—Ni1	125.51 (9)	H8A—C8—H8C	109.5
Ni1—O4—H41	122.0 (15)	H8B—C8—H8C	109.5
Ni1—O4—H42	97.1 (14)	N1—C9—C10	122.60 (14)
H42—O4—H41	108 (2)	N1—C9—H9	118.7
C9—N1—Ni1	123.09 (10)	C10—C9—H9	118.7
C13—N1—Ni1	118.67 (10)	C9—C10—C11	118.88 (14)
C13—N1—C9	118.21 (12)	C9—C10—H10	120.6
C14—N2—H21	122.3 (13)	C11—C10—H10	120.6
C14—N2—H22	117.1 (12)	C10—C11—C12	119.04 (14)
H21—N2—H22	118.3 (18)	C10—C11—H11	120.5
O1—C1—C2	118.42 (13)	C12—C11—H11	120.5
O2—C1—O1	124.67 (14)	C11—C12—C13	118.29 (13)
O2—C1—C2	116.86 (13)	C11—C12—C14	124.54 (13)
C3—C2—C1	121.08 (13)	C13—C12—C14	117.16 (13)
C3—C2—C7	118.83 (14)	N1—C13—C12	122.98 (13)
C7—C2—C1	119.91 (13)	N1—C13—H13	118.5
C2—C3—H3	119.8	C12—C13—H13	118.5
C4—C3—C2	120.46 (14)	O3—C14—N2	122.43 (14)
C4—C3—H3	119.8	O3—C14—C12	119.86 (13)
C3—C4—H4	119.6	N2—C14—C12	117.70 (13)
			
O4—Ni1—O1—C1	153.43 (11)	O2—C1—C2—C7	23.4 (2)
O4^i^—Ni1—O1—C1	−26.57 (11)	C1—C2—C3—C4	172.95 (13)
N1—Ni1—O1—C1	66.68 (11)	C7—C2—C3—C4	−2.1 (2)
N1^i^—Ni1—O1—C1	−113.32 (11)	C5—C4—C3—C2	1.0 (2)
O1—Ni1—N1—C9	−144.39 (11)	C3—C4—C5—C6	1.2 (2)
O1^i^—Ni1—N1—C9	35.61 (11)	C3—C4—C5—C8	−179.53 (14)
O1—Ni1—N1—C13	37.62 (11)	C4—C5—C6—C7	−2.3 (2)
O1^i^—Ni1—N1—C13	−142.38 (11)	C8—C5—C6—C7	178.42 (15)
O4—Ni1—N1—C9	128.90 (12)	C6—C7—C2—C1	−174.10 (14)
O4^i^—Ni1—N1—C9	−51.10 (12)	C6—C7—C2—C3	1.0 (2)
O4—Ni1—N1—C13	−49.10 (11)	C2—C7—C6—C5	1.2 (2)
O4^i^—Ni1—N1—C13	130.90 (11)	N1—C9—C10—C11	0.1 (2)
Ni1—O1—C1—O2	17.6 (2)	C12—C11—C10—C9	0.0 (2)
Ni1—O1—C1—C2	−159.77 (9)	C13—C12—C11—C10	0.1 (2)
Ni1—N1—C9—C10	−178.28 (11)	C14—C12—C11—C10	−178.64 (14)
C13—N1—C9—C10	−0.3 (2)	C11—C12—C13—N1	−0.3 (2)
Ni1—N1—C13—C12	178.46 (11)	C14—C12—C13—N1	178.56 (13)
C9—N1—C13—C12	0.4 (2)	O3—C14—C12—C11	178.48 (14)
O1—C1—C2—C3	26.1 (2)	O3—C14—C12—C13	−0.3 (2)
O1—C1—C2—C7	−158.94 (14)	N2—C14—C12—C11	−1.1 (2)
O2—C1—C2—C3	−151.54 (14)	N2—C14—C12—C13	−179.83 (13)

Symmetry codes: (i) −*x*, −*y*, −*z*+1.

### Hydrogen-bond geometry (Å, °)

**Table:**

Cg2 is the centroid of the N1/C9–C13 ring.

**Table:**

*D*—H···*A*	*D*—H	H···*A*	*D*···*A*	*D*—H···*A*
N2—H21···O2^ii^	0.86 (2)	2.037 (19)	2.8333 (18)	153.4 (19)
N2—H22···O3^iii^	0.90 (2)	2.05 (2)	2.9192 (19)	161.5 (18)
O4—H41···O3^iv^	0.81 (2)	2.10 (2)	2.8864 (16)	162.9 (19)
O4—H42···O2^i^	0.89 (2)	1.75 (2)	2.6240 (16)	165 (2)
C6—H6···Cg2^v^	0.93	2.65	3.5737 (18)	171

Symmetry codes: (ii) −*x*, −*y*+1, −*z*+1; (iii) −*x*+1, −*y*+1, −*z*+1; (iv) −*x*+1, −*y*, −*z*+1; (i) −*x*, −*y*, −*z*+1; (v) *x*, *y*, *z*+1.

## References

[bb1] Bigoli, F., Braibanti, A., Pellinghelli, M. A. & Tiripicchio, A. (1972). *Acta Cryst.* B**28**, 962–966.

[bb2] Bruker (2005). *SADABS* Bruker AXS Inc. Madison, Wisconsin, USA.

[bb3] Bruker (2007). *APEX2* and *SAINT* Bruker AXS Inc. Madison, Wisconsin, USA.

[bb4] Farrugia, L. J. (1997). *J. Appl. Cryst.* **30**, 565.

[bb5] Farrugia, L. J. (1999). *J. Appl. Cryst.* **32**, 837–838.

[bb6] Hökelek, T., Dal, H., Tercan, B., Özbek, F. E. & Necefoğlu, H. (2009*a*). *Acta Cryst.* E**65**, m466–m467. 10.1107/S1600536809011209 PMC2968958 21582397

[bb7] Hökelek, T., Dal, H., Tercan, B., Özbek, F. E. & Necefoğlu, H. (2009*b*). *Acta Cryst.* E**65**, m513–m514. 10.1107/S160053680901318X PMC2977573 21583759

[bb8] Hökelek, T., Dal, H., Tercan, B., Özbek, F. E. & Necefoğlu, H. (2009*c*). *Acta Cryst.* E**65**, m607–m608. 10.1107/S1600536809015645 PMC2977639 21583825

[bb9] Hökelek, T., Gündüz, H. & Necefoğlu, H. (1996). *Acta Cryst.* C**52**, 2470–2473.

[bb10] Hökelek, T. & Necefoğlu, H. (1998). *Acta Cryst.* C**54**, 1242–1244.

[bb11] Krishnamachari, K. A. V. R. (1974). *Am. J. Clin. Nutr.* **27**, 108–111. 10.1093/ajcn/27.2.108 4812927

[bb12] Sheldrick, G. M. (2008). *Acta Cryst.* A**64**, 112–122. 10.1107/S0108767307043930 18156677

[bb13] Spek, A. L. (2009). *Acta Cryst.* D**65**, 148–155. 10.1107/S090744490804362X PMC2631630 19171970

